# Hyperspectral Imaging and Chemometric Modeling of *Echinacea* — A Novel Approach in the Quality Control of Herbal Medicines

**DOI:** 10.3390/molecules190913104

**Published:** 2014-08-26

**Authors:** Maxleene Sandasi, IIze Vermaak, Weiyang Chen, Alvaro M. Viljoen

**Affiliations:** 1 Department of Pharmaceutical Sciences, Tshwane University of Technology, Private Bag X680, Pretoria 0001, South Africa; E-Mails: sandasim@tut.ac.za (M.S.); vermaaki@tut.ac.za (I.V.); chenw@tut.ac.za (W.C.); 2 Department of Pharmaceutics and Industrial Pharmacy, Faculty of Pharmacy King Abdulaziz University, Jeddah 21589, Saudi Arabia

**Keywords:** *Echinacea*, chemometrics, hyperspectral imaging, principal component analysis, partial least squares discriminant analysis, quality control

## Abstract

*Echinacea* species are popularly included in various formulations to treat upper respiratory tract infections. These products are of commercial importance, with a collective sales figure of $132 million in 2009. Due to their close taxonomic alliance it is difficult to distinguish between the three *Echinacea* species and incidences of incorrectly labeled commercial products have been reported. The potential of hyperspectral imaging as a rapid quality control method for raw material and products containing *Echinacea* species was investigated. Hyperspectral images of root and leaf material of authentic *Echinacea* species (*E. angustifolia*, *E. pallida* and *E. purpurea*) were acquired using a sisuChema shortwave infrared (SWIR) hyperspectral pushbroom imaging system with a spectral range of 920–2514 nm. Principal component analysis (PCA) plots showed a clear distinction between the root and leaf samples of the three *Echinacea* species and further differentiated the roots of different species. A classification model with a high coefficient of determination was constructed to predict the identity of the species included in commercial products. The majority of products (12 out of 20) were convincingly predicted as containing *E. purpurea*, *E. angustifolia* or both. The use of ultra performance liquid chromatography-mass spectrometry (UPLC-MS) in the differentiation of the species presented a challenge due to chemical similarities between the solvent extracts. The results show that hyperspectral imaging is an objective and non-destructive quality control method for authenticating raw material.

## 1. Introduction

**Figure 1:**
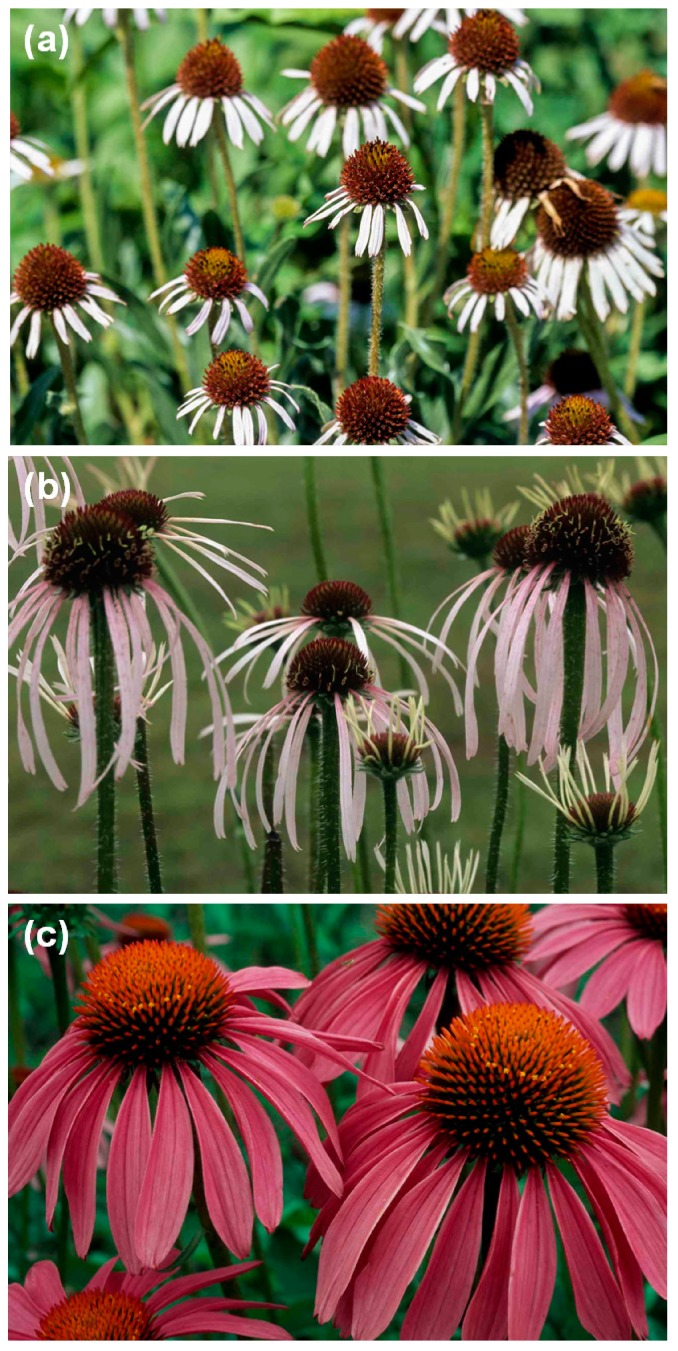
Photographs of the three *Echinacea* species: (**a**) *E. angustifolia*; (**b**) *E. pallida*; and (**c**) *E. purpurea*.

*Echinacea* species have a long history of use as traditional medicine to treat various ailments, including infections such as syphilis and septic wounds, cough, sore throat and tonsillitis. The three species currently used medicinally and most commonly to treat the common cold, influenza-like illnesses and upper respiratory tract infections (URTIs) are *Echinacea angustifolia* DC., *Echinacea pallida* (Nutt.) Nutt. and *Echinacea purpurea* (L.) Moench [1,2]. *Echinacea* species (Figure 1) have collectively been referred to as purple cornflower and pre-1968, *E. angustifolia* and *E. pallida* were considered to be varieties of the same species. *Echinacea pallida* is sometimes referred to as pale coneflower or pale purple coneflower and *E. angustifolia* is known as narrow leaf purple coneflower or Kansas snakeroot. A revision of the species suggested the following nomenclature; *E. pallida* var. *angustifolia* (DC.) Cronq. and *E. pallida* var. *pallida* (Nutt.) Cronq but the three species (*E. angustifolia*, *E. pallida* and *E. purpurea*) are still separately indicated on commercial products and will therefore be referred to as such in this study. Clinical trials to investigate the effectiveness of *Echinacea* preparations in the treatment of URTIs have reported effects superior to placebo, but the phytochemical diversity makes the interpretation of research findings difficult [1]. A structured review on clinical trials concluded that *Echinacea* use has no benefit in the treatment and prevention of colds [2]. Despite the publication of some negative research results, *Echinacea* products remain extremely popular, and are still frequently mentioned and/or promoted in the media and are commercially important. In the United States of America, annual sales of all *Echinacea* products exceeded $200 million in 2000 and 2001 whereafter it steadily declined to about $129 million in 2006 [3]. According to the Nutrition Business Journal, *Echinacea* sales rose to $132 million in 2009 [4] and it is still one of the best-selling herbal preparations in the USA [1].

There is variation in the chemical constituents of *Echinacea* between the different species and within different plant parts. The main chemical constituents include alkamides, phenylpropanoids, polysaccharides and volatile oils, as well as minor constituents such as flavonoids. The chemical composition, including alkamide content, determined using liquid chromatography, is traditionally used to establish the quality of plant material and preparations, as well as to identify the species [1]. According to a review by Barnes [1], several poor quality products have been identified, some not stating the species or plant parts used and others purporting to contain a certain species while in fact it does not. Of 59 commercial products analysed, 28 (48%) did not contain the species claimed on the label. Clearly, developing methods that can rapidly authenticate plant material will have a positive impact on the quality of products distributed to consumers. 

While the interchangeable use of the three *Echinacea* species in commercial products has not been reported as posing any potential risk, evidence of pharmacological equivalence of these species has not been clearly demonstrated. The phytochemical diversity in the raw materials (species) and commercial products that are widely available as directly pressed juices, ethanolic or hydrophilic extracts, powdered leaves, flowers and roots as well as extracts standardised to marker constituents such as phenolic acid or echinacoside content may raise concerns relating to efficacy [5]. Research on the efficacy of *Echinacea* raw materials and commercial products as immunomodulating agents has been conducted both *in vitro* and *in vivo*. Evidence of varying degrees of efficacy of *Echinacea* was demonstrated in mouse models where *E. purpurea* demonstrated superior immunomodulation activity compared to *E. angustifolia* and *E. pallida* [6]. In a separate study, *E. purpurea* whole powder again demonstrated consistently high macrophage activation, while products standardised to marker constituents were found to be inactive as immunomodulators [7]. A high degree of variability among similarly standardised extracts was also observed. A comprehensive review on the quality and pharmacological activities of *Echinacea* raw materials and commercial products reports on the inconsistencies in product quality while pre-clinical and clinical studies on many commercial products do not demonstrate pharmacological equivalence among products [3]. This variation between products and subsequent implications on efficacy should be considered important consumer information which may assist in the choice of product. To date however, there is no consensus with regards to the species, plant part or extraction methods that yields a product with the most desirable pharmacological activity.

**Figure 2:**
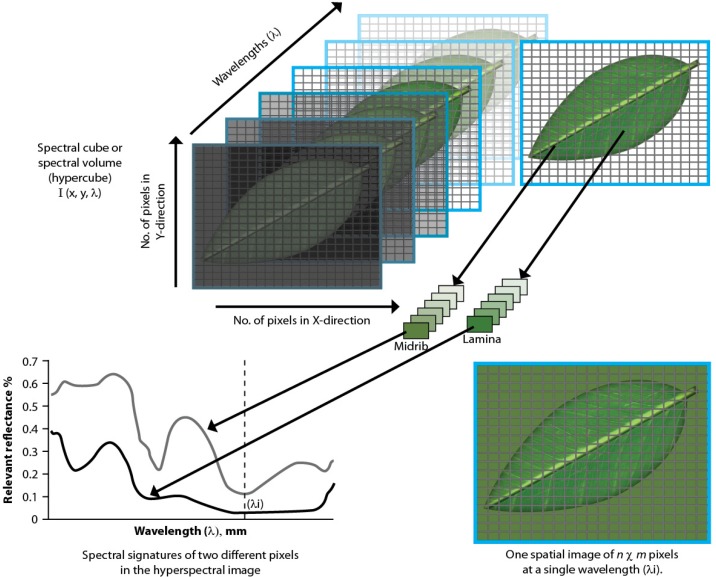
An illustration of a spectral hypercube created through the combination of two spatial (x;y) and one wavelength (λ) dimension in hyperspectral imaging.

Hyperspectral imaging (HSI) acquires both spectral and spatial information from a sample through a combination of conventional spectroscopy and imaging [8]. It has been used with success as an analytical tool to assess raw material and product quality in the agricultural, food and beverage and pharmaceutical industries amongst others [8,9], where non-destructive analyses can be performed in a much shorter time compared to conventional analysis methods such as liquid chromatography. In hyperspectral imaging, a hypercube is created through the combination of two spatial (x;y) and one wavelength (λ) dimension (Figure 2). Images are collected as a function of wavelength resulting in a stack of images referred to as a hypercube. At any wavelength, the image comprises of several pixels (2D-squares) where each pixel represents a spectrum containing point chemical information. The images are then analysed to identify wave regions where chemical differences are observed within the sample under investigation (Figure 2). The inclusion of spatial information yields more information about a sample and unknown samples can be more accurately predicted in some cases as compared to single-point spectroscopy. Multivariate analysis tools such as principal component analysis (PCA) and partial least squares (PLS) analysis are used to reduce the high dimensionality of data and to display compositional differences in an image enabling the development of models that can be used for quality control purposes [8,10,11].

The aim of this study was to investigate the potential of hyperspectral imaging in combination with multivariate data analysis to distinguish between the three commercially important *Echinacea* species and to correctly identify the *Echinacea* species present in commercial products.

## 2. Results and Discussion

### 2.1. Image Analysis Using PCA

**Figure 3:**
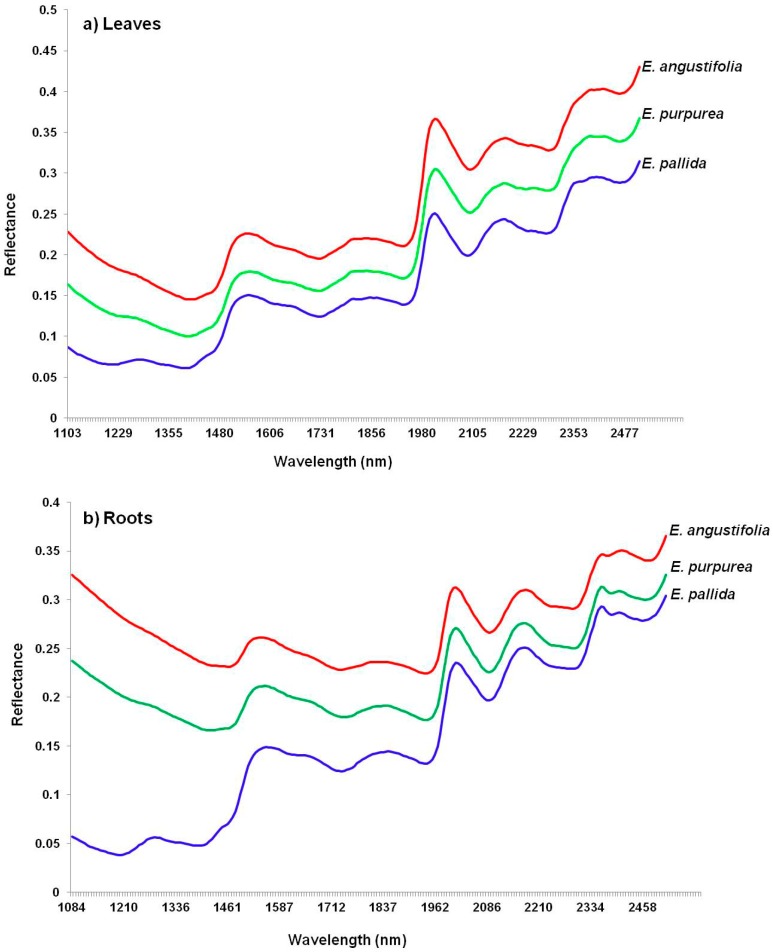
NIR imaging spectra of (**a**) *Echinacea* leaves and (**b**) roots showing similar spectral patterns of the three species.

Figure 3 displays NIR spectra of both leaf (a) and root (b) powders for the three *Echinacea* species. The spectral patterns do not show distinctive features that can be used to differentiate the species and hence the need for PCA which provides visual plots such as score images and scatter plots to observe clearer differences. 

**Figure 4:**
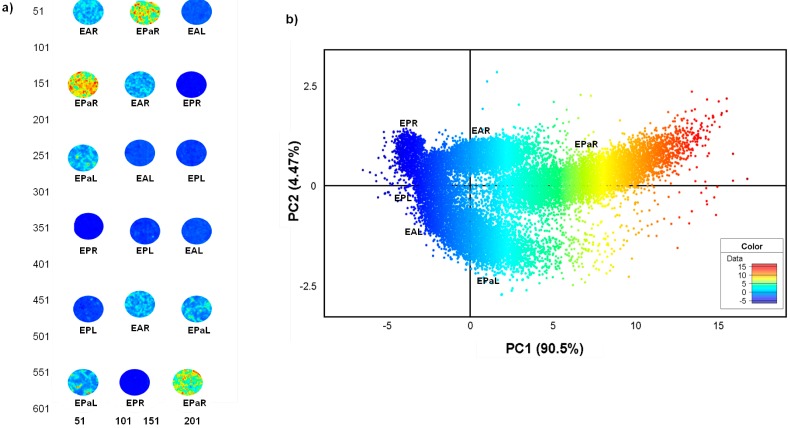
PCA score image (t1) of *Echinacea* root and leaf powders showing distinction of the three species based on colour amplitudes (**a**). The corresponding score plot (PC1 *vs.* PC2) shows distinct pixel clusters coloured according to score values that correspond to the images (**b**). (EAL—*E. angustifolia* leaf, EPL—*E. purpurea* leaf, EPaL—*E. pallida* leaf, EAR—*E. angustifolia* root, EPR—*E. purpurea* root, EPaR—*E. pallida* root).

The interactive score image and scatter plots were coloured according to the score values of PCs. The score image is an amplitude plot where similar colours represent similar score values and *vice-versa*. Principal component analysis of the root and leaf image showed a clear separation of *E. pallida* from the other two species based on the colour amplitude, as observed in the PCA score image of the first PC (t_1_) after mean centering and standard normal variate (SNV) correction was applied to the data (Figure 4a). Replicate *E. pallida* root samples (EPaR) showed a consistently distinct high colour amplitude (yellow-red) compared to the other species, evidence that its chemical profile differed significantly from the other species. As observed for the root powders, *E. pallida* leaf (EPaL) also showed a higher colour profile (light blue) compared to leaves of *E. angustifolia* (EAL) and *E. purpurea* (EPL) that had low amplitude of -5 (dark blue) (Figure 4a). 

The corresponding scatter plot shows pixels coloured according to score values which correlates to the amplitudes scale in the score image. Using PCA, chemical variation was observed within the HSI data which revealed 6 pixel clusters with each cluster representing plant parts of different species (Figure 4b). The cumulative chemical variation modeled using three principal components (PCs) was 97.2% (R^2^X_cum_ = 0.972). The variation of 90.5% along PC1 was responsible for separating mainly the root powders where *E. pallida* (EPaR) was shown to be the most distinct with its pixel cluster (highest score value-red yellow) falling on the far positive PC1. The *E. angustifolia* root cluster (EAR) showed pixels with lower score values spanning Y = 0 region, while *E. purpurea* root pixels (EPR) occupied negative PC1 (Figure 4b). The observation supports results of the score image where maximum variance modeled along PC1 was shown to differentiate mainly the root samples. Distinction of the leaf samples was observed along PC2 in the scatter plot; however, the variation appears minimal (4.47%) since the presence of root samples influenced variation within the model. 

**Figure 5:**
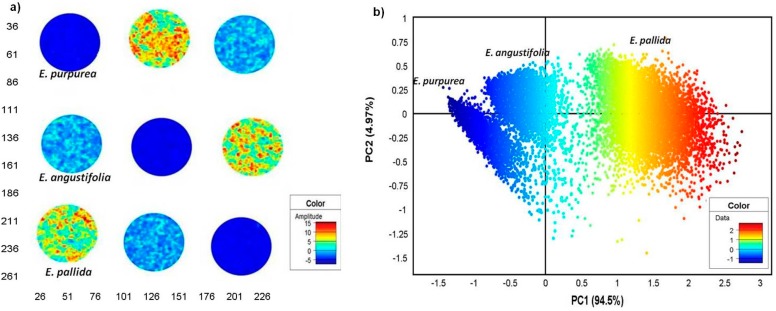
PCA score image (t1) of *Echinacea* root powders showing distinction of the three species based on colour amplitudes (**a**). The corresponding score plot (PC1 *vs.* PC2) shows distinct pixel clusters coloured according to score values that correspond to the images (**b**). (EAR—*E. angustifolia* root, EPR—*E. purpurea* root, EPaR—*E. pallida* root).

To obtain a clearer picture of the chemical distinction of the root samples, it was necessary to model these separately from the leaves to eliminate the influence of the leaf chemistry on the model. Figure 5a is a score image of PC1 which showed three distinct colour amplitudes, each representing a different species. As observed in Figure 4a, *E. pallida* displayed the highest colour amplitude (orange-yellow) while *E. angustifolia* (light blue) and *E. purpurea* (dark blue) showed lower amplitudes that differed slightly. The corresponding scatter 2D density plot demonstrated a clear separation of the three pixel clusters along PC1 with 94.5% chemical variation in the data cube attributed to the distinction of the three species (Figure 5b). 

**Figure 6:**
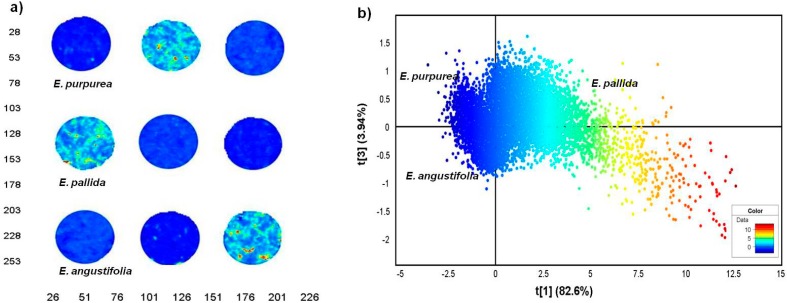
PCA score image (t1) of *Echinacea* leaf powders based on colour amplitudes (**a**). The corresponding score plot (PC1 *vs.* PC3) shows minimal separation of the pixel clusters (**b**). (EAL—*E. angustifolia* leaf, EPL—*E. purpurea* leaf, EPaL—*E. pallida* leaf).

**Figure 7:**
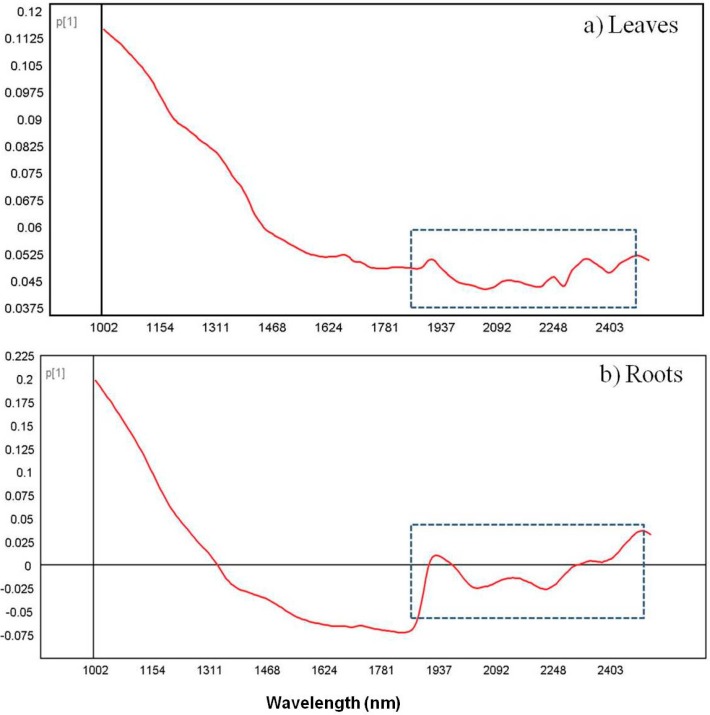
Loadings line plot of vector P1 for the leaf score image (**a**) and the root score image (**b**) showing variables responsible for separation of the three species.

The distribution of the pixel clusters along PC1 was consistent with Figure 4b, only clearer. The results for the root samples demonstrate that the three *Echinacea* species have distinct chemical profiles which was easily identified using NIR chemical imaging. To analyse the leaf data clearly, the leaf samples were modeled separately to exclude the influence from the roots. Figure 6a shows the score image where *E. pallida* displayed colour distinction (light blue-yellow) while similarities between *E. angustifolia* and *E. purpurea* leaves (dark blue) were evident. The corresponding scatter 2D plot of pixels demonstrates separation of the *E. pallida* cluster from the other two species along PC1. The majority of the variance was captured in the first two PCs with PC1 accounting for 82.6% of the data and 3.94% in PC2 (Figure 6b). It is clear from the plots that the leaf chemistry of the three species is less varied as demonstrated by the variance parameters. However, *E. pallida* demonstrated chemical distinction compared to the other species as identified by HSI and multivariate data analysis techniques. To investigate the differences observed in the score images of the leaves and roots, loadings line plots of the first vector (P1) were constructed for the leaf model (Figure 7a) and the roots model (Figure 7b). The loadings plots show the region between 1937–2400 nm as carrying discriminating information of the three species based on both the leaf and the root chemistry. In both cases, positive (peaks) and negative loadings (troughs) were recorded in this region and these regions could be further investigated for molecular signals that can be assigned to specific plant species. 

Based on these observations, it is evident that the use of NIR chemical imaging for the qualitative differentiation of *Echinacea* species presents a promising visual technique in the quality control of the raw material. The root chemistry of the species presents a better choice for analysis; however, the minor chemical differences between leaf samples can also be detected using the imaging tool. Previous reports on the chemical profiling of *Echinacea* species have reported marked differences between *E. angustifolia* and *E. pallida* that were previously regarded as varieties of the same species until they were taxonomically revised in 1968 [12]. In this study, *E. pallida* has demonstrated a distinct chemical profile that could be observed in both root and leaf samples.

**Figure 8:**
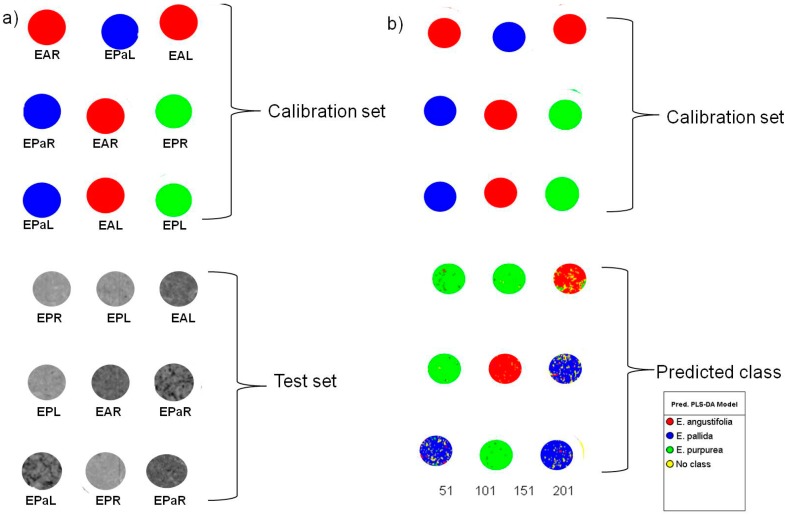
PLS-DA Y-image showing calibration samples used to build the model and test samples that were excluded for model validation (**a**). The predictions show test samples assigned to classes that correspond to the species imaged (**b**) (EAL—*E. angustifolia* leaf, EPL—*E. purpurea* leaf, EPaL—*E. pallida* leaf, EAR—*E. angustifolia* root, EPR—*E. purpurea* root, EPaR—*E. pallida* root).

Having successfully established that HSI can distinguish between the three *Echinacea* species, the next objective was to develop a PLS-DA model to predict the identity of commercial *Echinacea* products introduced into the model as an external dataset. Since many *Echinacea* products are prepared using both the leaf and root materials, a model that included both the leaves and the roots was constructed. Ultimately the model predictions would then identify the species present but not specify whether the material used was the root, leaf or both. Figure 8a is a PLS 1 Y-image of the PLS-DA model where the calibration set showed each species (class) assigned a different colour while the background or some unknown areas were classified as “no class” implying that the sample/region within the image could not be correlated to the modeled data. The test set samples (grey-scale) were excluded for external validation of the model where the correct identities were known. Introducing the external test set into the PLS-DA calibration model provided results shown in Figure 8b. The predicted classes of the test samples were in agreement with prior knowledge on identities of the samples based on the colour coding. There were however regions that were misclassified within the samples which can be attributed to chemical similarities between the species and hence the overlap in the pixel data. The PLS-DA model however exhibited good model statistics with R^2^X_cum_ of 0.980 and cumulative variation of Y (Q^2^Y_cum_) of 0.779 that could be predicted by three components which was subsequently used for class prediction of external samples.

### 2.2. Class Prediction of Commercial Echinacea Samples

**Figure 9:**
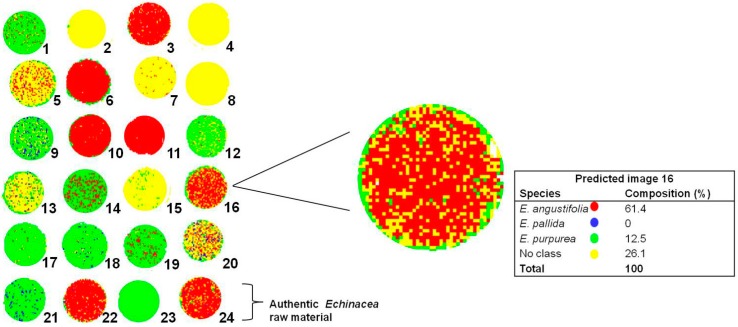
PLS prediction images (t_1_) of *Echinacea* commercial products and authentic raw material showing variation in the chemical composition of *Echinacea* commercial products. The enlarged insert demonstrates the predicted levels of each species in product 16.

Twenty commercial *Echinacea* samples and four authentic *Echinacea* raw material control samples captured as a single image were introduced into the PLS-DA model for class prediction. Figure 9 shows the PLS-DA prediction image after matching the chemical profiles of the products to the authentic *Echinacea* samples. 

The predictions are represented by colour where *E. angustifolia* is represented in red, *E.* *pallida* in blue and *E. purpurea* in green. The results indicate that 12 out of 20 products were correctly classified (indicated with ^a^) with the HSI class prediction matching the product label. The remaining eight products were misclassified (indicated with ^b^) as the HSI prediction did not match the product label. Seven of the 20 products contained high levels of *E. purpurea* (1, 9, 12, 14, 17, 18 and 19) while five products contained high levels of *E. angustifolia* (3, 6, 10, 11 and 16). Five of the 20 products (2, 4, 7, 8, and 15) seemed to present a completely different profile to the powdered authentic *Echinacea* species and hence these were identified as no class (yellow). Upon investigation it was discovered that most of the samples predicted as ‘no class’ were either labeled as extracts or concentrates of *Echinacea* and not unprocessed raw material. As chemical processing alters the chemistry of the products, this presents chemical variation between the modeled and predicted samples and hence the products were not classified as *Echinacea*. In some images (5, 13 and 20) the ‘no class’ prediction was more prominent while trace amounts of one or two *Echinacea* species was detected. The observation can be explained by the presence of excipients such as magnesium stearate in the formulations in greater proportions compared to *Echinacea* raw material. 

Multi-ingredient formulations containing other herbs such as garlic, parsley and goldenseal were also predicted as containing trace amounts of the *Echinacea* species while the majority of the constituents could not be classified (Product 20). The last sample row in the image containing authentic *Echinacea* raw material as a control demonstrates the accuracy of the model in predicting *Echinacea* raw material. The four samples (21, 22, 23 and 24) were correctly identified where the major species predicted to be dominant in the sample matched the species imaged (root and/or leaf samples). The potential of HSI in determining the presence or absence of *Echinacea* raw material in both commercial products and raw material samples has been demonstrated.

**Table 1:** Results of the HSI classification analysis using the (PLS-DA) model in comparison to the product label.

Product	Product Label Claim	HSI Species Predictions (%)			
		*E. angustifolia*	*E. purpurea*	*E. pallida*	No Class
1	*E. purpurea* (herb, root) & *E. angustifolia* herb	1.9	89.8 ^a^	0	8.3
2	*E. angustifolia* root extract	0	0	0.2	99.8 ^b^
3	*E. purpurea* root *&* aerial parts	89.3 ^a^	2.6	0.2	7.9
4	*E. purpurea* Herba & Radix dry concentrate	0	0	0	100 ^b^
5	*E. purpurea & E. angustifolia*	14.3	7.7	1.2	76.8 ^b^
6	*E. purpurea* root & *E. angustifolia* root	94.6 ^a^	5.4	0	0.1
7	*Echinacea purpurea* extract	0	0	0	100 ^b^
8	*E. purpurea* Herba & *E. purpurea* Radix	0	0	0	100 ^b^
9	*E. pallida*	0	72.2 ^a^	7.4	20.4
10	*E. purpurea* root & *E. angustifolia* root	86.7 ^a^	12.6	0	0.7
11	*E. angustifolia* root	99.5 ^a^	0	0.1	0.4
12	*E. purpurea* herb	0.2	73.2 ^a^	0.2	26.4
13	*E. pallida* (outer label) & *E. purpurea* (inner label)	1.3	22.9	2.4	73.3 ^b^
14	*Echinacea* aerial powder & root powder	9.2	90.1 ^a^	0	0.7
15	*E. angustifolia* root & rhizome	0	0	0	100 ^b^
16	*E. purpurea* stem, leaf, flower	61.8 ^a^	12.1	0	26.1
17	*E. purpurea* root	0.2	95.4 ^a^	0	4.4
18	*Echinacea* blend (*angustifolia*, *pallida*, *purpurea*)	0.1	92.4 ^a^	2	5.6
19	*Echinacea* leaf powder & standardised extract	4.9	88.5 ^a^	0	6.6
20	*Echinacea*, Goldenseal, Elderberry, Garlic & Parsley	16.3	8.1	9.4	66.3 ^b^
21	*E. purpurea* leaf (authentic raw material)	0.1	90.2 ^a^	6.5	3.3
22	*E. angustifolia* root (authentic raw material)	89.1 ^a^	3.5	0.4	7
23	*E. purpurea* root (authentic raw material)	0	97.5 ^a^	0	2.5
24	*E. angustifolia* leaf (authentic raw material)	64.2 ^a^	6.4	1.8	27.5

^a^ The formulation was predicted to contain the indicated species in highest proportions. ^b^ The formulation was predicted to contain little or no authentic *Echinacea* raw material.

In addition to a qualitative assessment of the product composition, it was also possible to predict the percentage composition of the constituent species within a product by highlighting the species in the image and obtaining the corresponding prediction table as demonstrated in Figure 9 (insert). The quantitative predictions are reported in Table 1 together with a comparison of the product label. Table 1 shows that most of the commercial products does contain *Echinacea* raw material in high levels and most of the HSI results are in agreement with the label claim. However, HSI analysis detected the presence of more than one species in many of the products. According to Table 1, *E. purpurea* and *E. angustifolia* seem to be the raw materials of choice in many products while *E. pallida* is rarely used. Only two products were labeled as containing *E. pallida*; however, according to the predictions, *E. purpurea* was identified as occurring in higher proportions in both cases while the presence of *E. pallida* was almost negligible (<10%). 

### 2.3. Ultra Performance Liquid Chromatography-Mass Spectrometry (UPLC-MS) Analysis

**Figure 10:**
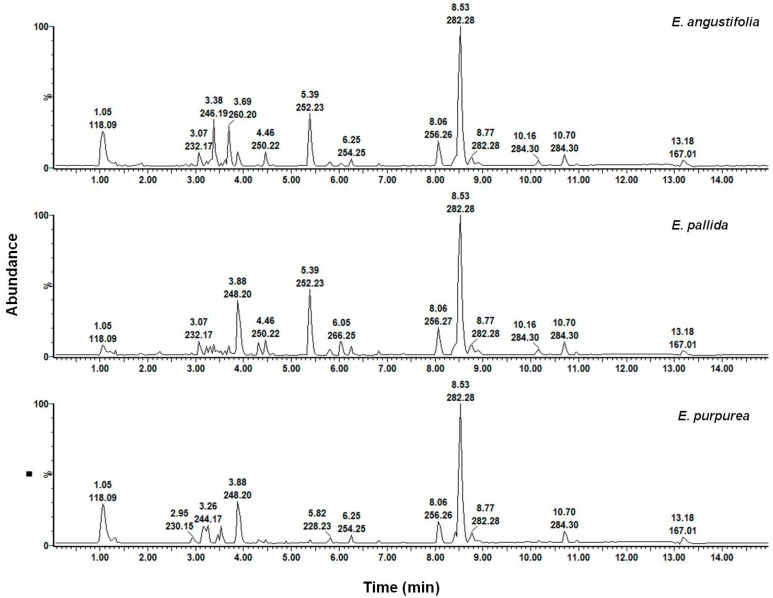
Chromatographic fingerprints of methanol root extracts of *Echinacea* species.

In order to confirm the HSI predictions, UPLC-MS was used to analyse both the raw materials and the products to determine the botanical species in the products. Three solvent systems (methanol, hexane and chloroform), previously reported to provide some distinction between the three *Echinacea* species, were used. Only the methanol and hexane root extracts were used for product classification as the leaf chromatograms did not display any clear distinction between the three species. Additionally, the chloroform root profiles were very similar to that of hexane, therefore only the hexane chromatograms will be presented for discussion. The BPI chromatograms of each product were qualitatively assessed against the chromatograms of the three different species to identify similarities or differences in the chemical profiles, but marked chemical similarities of the solvent extracts of the three species were observed. Figure 10 shows the BPI chromatograms of the methanol root extracts for the authentic raw materials. The chromatograms demonstrate marked similarities between the three species with the major peaks present in all three species.

**Figure 11:**
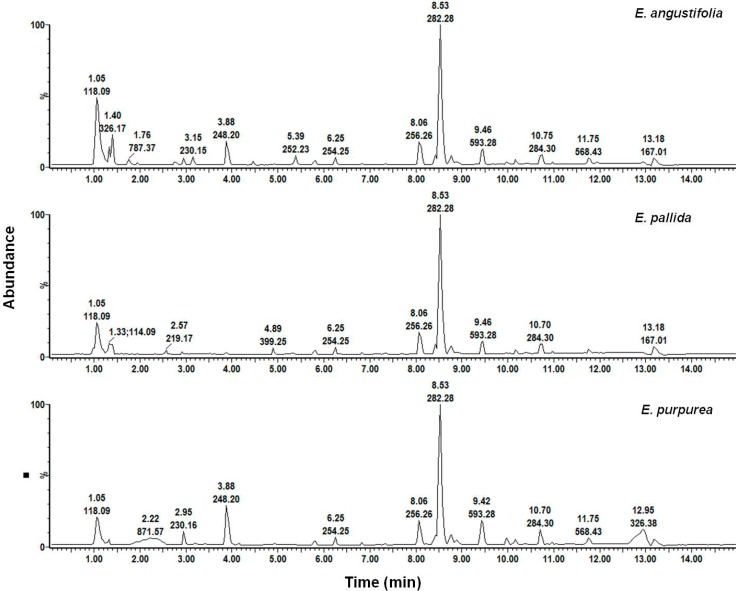
Chromatographic fingerprints of methanol leaf extracts of *Echinacea* species.

An inspection of the methanol leaf chromatograms (Figure 11) showed similarities in the major peaks, thus both methanol and hexane root extracts presented a challenge in their usefulness to differentiate between the three species. Closer inspection of the methanol chromatograms revealed that minor peaks in the region between 3–5 min appeared to be unique to a species and were therefore selected for qualitative assessment. The methanol chromatogram of product 9, which was labeled as *E. pallida* but predicted as *E. purpurea*, was compared to the chromatograms of the authentic material.

**Figure 12:**
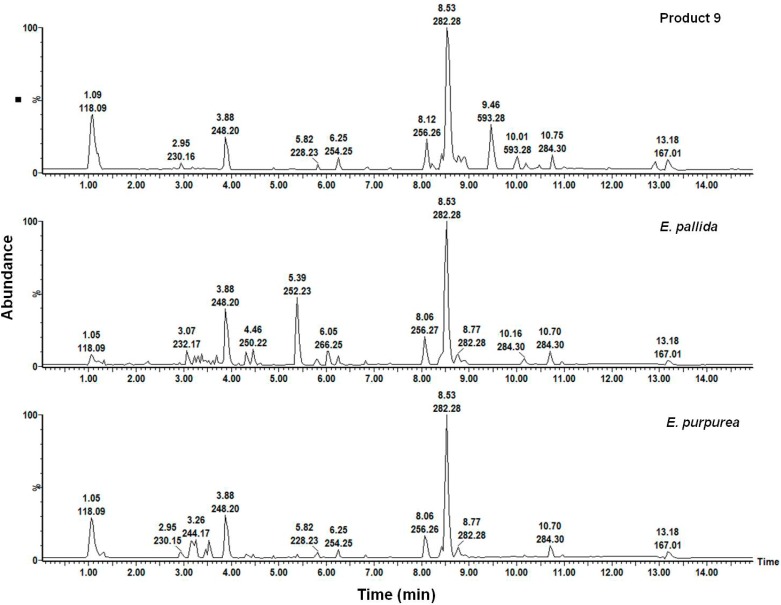
A comparison of the methanol root extracts of product 9 and two *Echinacea* species.

Figure 12 shows that product 9 was devoid of a signal present in *E. pallida* at Rt 5.39 min (*mz*^−1^ = 252.23). Additionally, a minor characteristic peak unique to *E. purpurea* at Rt 4.89 min (*mz*^−1^ = 277.22) is also present in product 9. Based on this comparison it was possible to confirm the HSI prediction that product 9 contains *E. purpurea* instead of *E. pallida* as claimed on the product label. The shortfalls of using UPLC-MS as a tool for the qualitative differentiation of *Echinacea* species and classification of commercial products were demonstrated due to the chemical similarities of the solvent extracts obtained from the raw materials. On the other hand, because HSI presents chemical profiles of the whole metabolome in an untargeted approach, it allows for any possible differences in the composition of the raw material to be detected using this approach in a simple manner.

Chemical maps in the form of colour images provide a visual assessment of the composition of the raw materials and the products. Where multiple constituent species are present, it is also easy to detect. This study also highlighted labeling errors as previously reported. The outer packaging of product 13 stated that *E. pallida* was used. The same product had an inner package insert that stated that *E. purpurea* was used. This product contained 22.4% of *E. purpurea* according to HSI prediction. Product 18 purported to contain an *Echinacea* blend with all three species. The HSI results revealed 92.4% *E. purpurea*, 0.1% *E. angustifolia* and 2.0% *E. pallida*. The question then arises: can this truly be described as a blend if it contains only 0.1% and 2.0% of the species which are considered part of the blend? A blend has not been defined and it is clear that specifications for herbal products need to be set and enforced. However, in order to achieve this, good quality control methods such as HSI need to be developed and implemented. 

## 3. Experimental Section

### 3.1. Sample Preparation

American Herbal Pharmacopoeia^®^ (AHP)-certified reference material (root and leaf specimens) of *E. angustifolia*, *E. pallida* and *E. purpurea* were purchased from the American Herbal Products Association (AHPA, Silver Spring, MD, USA). Twenty commercial products claiming to contain *Echinacea* were purchased from local retail outlets in South Africa, as well as international suppliers. Both the botanical reference standards (roots and leaves) and products (capsule contents and tablets) were powdered to attain homogeneity using a Retsch^®^ 400 MM ball mill (Haan, Germany) at a frequency of 30 Hz for 30 s. The powders were sieved through a 500 µm sieve (Endecotts Ltd., London, England) to ensure uniform particle size distribution and filled into plastic containers with a diameter of 8 mm. The surface of the powdered standards (in triplicate) and products were leveled with a spatula to minimise surface effects. 

### 3.2. Short Wave Infrared (SWIR) Hyperspectral Image Acquisition

Powdered botanical standards of both the roots and leaves of three *Echinacea* species were replicated three times to include a total of 18 samples, positioned randomly on a stage, and image acquisition commenced as the samples entered the field of view. The images were acquired using a sisuChema short wave infrared (SWIR) hyperspectral pushbroom imaging system (Specim, Spectral Imaging Ltd., Oulu, Finland) with Chemadaq version 3.62.183.19 software. The system consisted of an imaging spectrograph coupled to a 2-D array mercury-cadmium-telluride (HgCdTe) detector with a light source of quartz halogen lamps mounted in reflector housing. A 50 mm high magnification lens with a spatial resolution of 0.30 µm was used to capture images at a frame rate of 100 Hz at an exposure of 3.0 ms with a spectral range of 920–2514 nm and a resolution of 6–7 nm. Images with 256 × 320 pixels and a pixel depth of 14 bits/pixel were obtained. Following image capture of the standards, twenty commercial products were positioned on the stage and imaged separately under the same conditions at room temperature. Internal dark and white reference standards were used for image calibration and to correct for variation in sample illumination. 

### 3.3. SWIR Image Analysis

#### 3.3.1. Principal Component Analysis (PCA)

The raw images were corrected automatically for white and dark references and converted to pseudo-absorbance (A/D converter counts to absorbance) using Evince multivariate analysis software version 2.4.0 (UmBio AB, Umeå, Sweden) [10]. Principal component analysis (PCA), which is the first step in multivariate data analysis, was applied with mean centering on the whole image (including background). PCA reduces dimensionality of large datasets by decomposing interrelated variables into a new set of coordinates (PCs) that are uncorrelated and ordered in a way that the first few PCs account for most of the variation in the data [13,14]. The greatest variance in the X data observed was between the sample and the background (the stage). The interactive score image and scatter density plots were used to remove pixels corresponding to the background, dead pixels and edge effects [10,11], revealing the variation between samples. Different mathematical pretreatment methods (standard normal variate (SNV), multiplicative scatter correction (MSC) and derivates) that minimise variability unrelated to the chemical composition of the powders were investigated for spectral pre-processing [15]. The method that provided the best separation with pertinent image differences in the score plot was chosen. The optimum number of principal components (PCs) was determined by excluding all PCs that did not significantly increase the value of the Q^2^Y_cum_. The regions at the beginning and at the end of NIR spectra are usually uninformative. Modeling these regions masks useful chemical information and hence the need to investigate these and remove them from the model so that clear chemical differences can be observed. In this study, the wavelengths ≤ 996 nm at the beginning of the spectra (920–996 nm) did not contain differentiating chemical information and it was excluded from the dataset resulting in better pixel classification. The resulting image was then assessed for chemical differences and similarities with the aim of differentiating between leaf and root samples of the different species.

#### 3.3.2. Partial Least Squares Discriminant Analysis (PLS-DA)

PLS-DA is a pattern recognition technique that correlates variation in the X data matrix (spectral data) to an independent Y-variable (class membership), which is categorical, resulting in the discrimination or classification of samples. This is a supervised classification technique as data is assigned into classes based on prior knowledge for effective prediction of group membership in new samples. In this study, the Y-variable was generated by selecting the leaf and root images of a species in the PCA score image, and assigning these to a class (Class A: *E. angustifolia*, Class B: *E. purpurea*, Class C: *E. pallida*). Half of the samples (*n* = 9) in the PCA score image consisting of 18 samples was assigned to classes and thus comprised the calibration set while the other half (*n* = 9) formed the test set. Cross-validation of the model was performed using a random selection method. Seven rounds (iterations) of cross-validation were performed as default settings in the software. PLS factors were added to the model and the optimum number was determined by excluding factors that did not significantly increase the value of the Q^2^Y_cum_ in the model. The PLS-DA score image (Y image) was then used for membership/class prediction (Min Y cut-off: 0.5; Max Y cut-off: 1.5) of the test set, mixtures and commercial samples. 

### 3.4. Ultra Performance Liquid Chromatography-Mass Spectrometry (UPLC-MS)

Three different solvents (methanol, chloroform and hexane) were used separately for extraction of both the leaf and root materials for UPLC-MS analysis. The extracts were then dissolved in super purity methanol to obtain a concentration of 5 mg/mL before injection into the UPLC-MS. The analysis was performed on a Waters Acquity Ultra Performance Liquid Chromatographic system equipped with a PDA detector (Waters, Milford, MA, USA). Separation was achieved on an Acquity UPLC BEH C_18_ column (150 mm × 2.1 mm, i.d., 1.7 μm particle size, Waters) maintained at 40 °C. Some preliminary tests were performed prior to setting the chromatographic conditions to obtain chromatograms with better resolution and a short analysis time. The mobile phase consisted of 0.1% formic acid (solvent A) and acetonitrile (solvent B) at a flow rate of 0.3 mL/min. The gradient elution was performed as follows: 61% A: 39% B changed to 30% A: 70% B in 2 min, to 20% A: 80% B in 4 min, to 10% A: 90% B in 4.5 min, maintained for 1 min and back to initial ratio in 1 min. The total run time was 15 min and the injection volume was 1.0 μL (full-loop injection). The positive and negative ion modes were examined and the positive ion mode provided results with more information and higher sensitivity. Thus, the mass spectrometer was operated in positive ion electrospray mode and nitrogen (N_2_) was used as the desolvation gas. Data were acquired between 100 and 1000 *mz*^−1^. The desolvation temperature was set to 350 °C at a flow rate of 500 Lh^−1^ and the source temperature was 100 °C. The capillary and cone voltages were set to 3500 and 35 V, respectively. Masslynx^®^ version 4.1 software was used to process and obtain all the chromatographic data. The base peak intensity (BPI) chromatograms of the botanical reference standards and commercial products were compared to verify the species contained in the commercial products based on MS spectra and retention time. This was performed to validate the results obtained using the HSI method.

## 4. Conclusions

The results revealed that hyperspectral imaging in combination with multivariate data analysis could convincingly distinguish between the three *Echinacea* species. In addition, the constructed PLS-DA model with a high coefficient of determination, accurately predicted the *Echinacea* species contained in several commercially available products and identified products that did not contain crude *Echinacea* raw material. Products that contained a mixture of species were also confirmed using HSI. The method is clearly more suited for raw material identification and sensitivity was limited in the prediction of polyherbal formulations or products containing a high level of excipients. It is evident that rapid quality control methods such as developed in this study are of utmost importance to ensure high quality products. The species used should always be specified on the label and correspond to the species that is present in the product. Clearly, hyperspectral imaging is suited for the quality control of herbal medicines as it is a rapid, non-destructive method with high prediction ability, which considers a large part of the phytochemical metabolome. 
